# Interpersonal Violence in Belgian Sport Today: Young Athletes Report

**DOI:** 10.3390/ijerph191811745

**Published:** 2022-09-17

**Authors:** Tine Vertommen, Mieke Decuyper, Sylvie Parent, Aurélie Pankowiak, Mary N. Woessner

**Affiliations:** 1 Forensic Psychology Research Unit, Thomas More University of Applied Sciences, 2018 Antwerp, Belgium; 2 Social Epidemiology and Health Policy, University of Antwerp, 2610 Antwerp, Belgium; 3 International Research Network on Violence and Integrity in Sport (IRNOVIS), 2610 Antwerp, Belgium; 4 Interdisciplinary Research Center on Intimate Relationship Problems and Sexual Abuse (CRIPCAS), C.P. 6128, Montréal, QC H3C 3J7, Canada; 5 Research Chair in Security and Integrity in Sport, Quebec City, QC G1V 0A6, Canada; 6 Department of Physical Education, Faculty of Education, Université Laval, Quebec City, QC G1V 0A6, Canada; 7 Institute for Health and Sport (IHES), Victoria University, Melbourne, VIC 3021, Australia

**Keywords:** violence, young athletes, sport, self-report, questionnaire, magnitude

## Abstract

Initiatives to safeguard athletes from interpersonal violence (IV) are rapidly growing. In Belgium, knowledge on the magnitude of IV in sport is based on one retrospective prevalence study from 2016 (*n* = 2.043 adults), involving those who had participated in organized sport for up to 18 years. Data on victimization rates in current youth sport populations are lacking. This study aimed to investigate the magnitude of IV in a sample of 769 athletes (aged between 13 and 21), using the Violence Towards Athletes Questionnaire (VTAQ). All types of IV were prevalent in this sample, ranging from 27% (sexual violence) to 79% (psychological violence and neglect). Boys reported significantly more physical violence, while girls reported significantly more sexual violence. IV perpetrated by peer athletes was reported to the same degree as IV perpetrated by a coach (70%), while IV perpetrated by a parent in the context of sport was somewhat less common, but still prevalent (48%). These findings, including factors associated with elevated exposure rates, can serve as a baseline measurement to monitor and evaluate current and future safeguarding interventions in Belgian sport.

## 1. Introduction

Athletes around the globe are stepping forward and speaking out against years of experiences of interpersonal violence (IV) in sport. After public disclosures of child sexual abuse by coaches (UK and Netherlands), and the testimonies of psychologically, physically, and sexually abusive training regimes in American gymnastics, several Belgian elite and amateur athletes found their voice and shared similar experiences e.g., [1,2]. The existence of IV against children, youth and adults in sport can no longer be ignored or denied and athlete protection and safeguarding policies, and their evaluation, are paramount. Measuring and monitoring the magnitude of violence against athletes in sport is a critical step to measuring the effectiveness of prevention efforts and policies.

Violence is a contested term and has numerous definitions. Operationalizations of the concept of violence vary worldwide. For our study we adopted the definition of violence as documented in article 19 of the Convention on the Rights of the Child [3]: *“[…] all forms of physical or mental violence, injury and abuse, neglect or negligent treatment, maltreatment or exploitation, including sexual abuse while in the care of parent(s), legal guardian(s) or any other person who has the care of the child”.*

Interpersonal violence (IV) involves the intentional use of physical force or power against other persons by an individual or small group of individuals [4]. Interpersonal violence may be physical, sexual, or psychological, and it may involve deprivation and neglect. Acts of IV can be further divided into family or partner violence and community violence. When studying IV in sport, one needs to distinguish behavior deemed to be a normal part of the game from that considered to overstep the ethical mark [5], i.e., deliberate or non-accidental IV. Accordingly, violence occurring within the bounds of prescribed constitutive rules is not considered in this study. This study focuses on IV perpetrated by peer-athletes, coaches and parents in the context of sport.

Initial studies, dating from the early 2010s, estimated the prevalence of IV in sport to vary between 23 and 79% for psychological violence, between 7 and 28% for sexual violence, and between 9 and 40% for physical violence [6,7,8,9,10,11]. The range of these estimates is broad. in part due to the lack of validated measurement tools, but also due to the use of different methodologies, sampling and operationalizations of the IV concept [12].

The first prevalence study into IV against child athletes in Belgium collected data from over 2000 Flemish adults who participated in youth sport [10]. The study showed that 38% of Belgian respondents experienced psychological violence, 17% sexual violence, and 14% physical violence in sport before the age of 18. The study further found that international level athletes, LGBT (Lesbian Gay Bisexual Transgender) athletes, athletes with a disability and athletes from an ethnic minority group reported higher levels of exposure to IV in sport.

Following the publication of these findings, Flemish sport authorities developed an integrity policy framework for all Flemish sport federations. In 2017, after several athletes disclosed their experiences with child sexual abuse perpetrated by their coaches, these policies were refined and strengthened, including the installation of local welfare officers, codes of conduct, awareness campaigns and educational programs for athletes, coaches and managers (Muyters, 2018). All of these initiatives are based on the assumptions of the magnitude of the problem on the only available prevalence study [10]. In 2018, one other study estimated the prevalence of IV against children in Flemish sport, as part of a broader aim to study violence against children in all areas [13]. The study found lower prevalence estimates than those found in Vertommen et al.’s study, but it is challenging to compare estimates from these two studies, due to significant differences in methodology. Van Haeken et al. [13] used the International Society for the Prevention of Child Abuse and Neglect Child Abuse Screening Tool-Child Institutional ICAST-CI [14]; that did not include all types of IV in sport. No other prevalence study into IV against child athletes has been conducted in Flemish sport since, meaning that base rates are currently lacking, and longitudinal monitoring and evaluation of the prevention initiatives cannot be initiated.

As noted, one of the challenges with collecting data on IV, particularly data that is comparable between nations, is the lack of a validated and widely used tool to measure IV in sport. However, the Canadian research group of Parent and colleagues recently developed the Violence Towards Athletes Questionnaire (VTAQ). It is the first validated questionnaire to measure violence towards young athletes [15]. The instrument is designed to survey young athletes’ experiences of all types of IV (neglect/psychological, physical and sexual violence) perpetrated by peer athletes, coaches and parents. The VTAQ was implemented in a sample of 1055 Quebecois young athletes between 14 and 17 years old, and resulted in a prevalence estimate of 79% for psychological violence, 40% for physical violence and 28% for sexual violence [9].

In addition to gathering data on prevalence of IV in sport, many studies are also seeking to understand what demographic and sport participation characteristics may predispose someone to experiencing higher rates of the various types of IV in sport. Most studies have explored aspects, such as age, sex, sexuality, sport specialization, competition level and hours of sport participation [9,10,11,16]. The results are incredibly mixed between the studies, however, with some suggesting being female is a risk factor for psychological violence and sexual violence, and others noting no sex differences. On the other hand, most studies found that being male is a risk factor for physical violence. Only the recent Canadian study by Willson and colleagues (2021) found higher exposure rates for physical violence in females. One explanation for this difference could be that Willson and colleagues included non-contact forms of physical violence (i.e., being forced to take doping) in physical violence, while studies using the VTAQ consider this psychological or instrumental violence, since these behaviors do not implicate a physical act [9]. Several studies have indicated that either a higher level of competition, early sport specialization or high weekly training hours are risk factors for psychological violence. The clearest agreement amongst the studies, however, is in the risk factors for sexual violence, with all four prevalence studies indicating that a non-heterosexual sexuality was associated with higher rates of sexual violence. Additionally, Vertommen et al. (2016) and Willson et al. (2021) found associations with ethnic minority and disability.

Recent information on the magnitude of IV in Belgian youth sport is lacking. The primary objective of the present study is to describe the magnitude of IV experienced by young recreational sport participants and competitive athletes between 13 and 21 years of age in sport today in Flanders (Belgium). In this study, we use the word ‘athletes’ to describe both competitive and recreational sport participants. The second objective of this study is to examine associations between these different types of IV (i.e., psychological-neglect, instrumental, physical, and sexual violence, as well as violence from a peer athlete, violence from a coach and violence from a parent) and factors that were previously associated with elevated exposure to IV, e.g., age, sex, ethnicity, sexual orientation, disability, early sport specialization, weekly training hours, sport level, and sport type.

## 2. Materials and Methods

### 2.1. Instrument

The Violence Toward Athletes Questionnaire (VTAQ) [15] is a self-report instrument to survey experiences of young athletes (14–17 years old) with all types of IV perpetrated by peer athletes, coaches and parents in the context of sport. The questionnaire consists of 70 items assigned to three sub domains (athlete, coach, parent perpetration) and consists of a detailed description of sport related violent behaviors of a psychological, negligent, physical and sexual nature. Items are scored on a 4-point scale from 0 (never), 1 (seldom; one or two times) and 2 (sometimes; 3 to 10 times), to 3 (often; more than 10 times). The original Canadian-French questionnaire was translated and validated in Dutch [17], which revealed an additional type of IV perpetrated by a coach, labelled ‘instrumental violence’. This type consists of experiences related to performance, e.g., being forced by a coach to: do additional trainings, to train while injured, to injure an opponent, to use performance-enhancing supplements, to lose weight or stop school. The Dutch version of VTAQ instrument showed good internal consistency on all factors in our sample: with Cronbach’s alpha ranging between 0.78 and 0.88 for IV perpetrated by an athlete, coach or parent, and between 0.76 and 0.86 for the broad types of violence (instrumental, psychological-neglect, physical, sexual) [17]. For this study, the total scores and domain scores per type of violence were examined as dichotomous variables using a low threshold measure to categorize athletes with at least one experience with violence (1) versus athletes with no experience (0).

Similar to the Canadian study, socio-demographic questions were included at the start of the questionnaire and allowed us to gather data on the age, sex, ethnic background, sexual orientation and participation in disability sport of the participating athletes. More information was gathered about their current or previous sport participation: type of sports, hours of weekly practice, highest sport level achieved and early sport specialization. To determine early single-sport specialization, defined as intense training in a single sport more than eight months per year before 12 years of age [18], three questions were presented: Before the age of 12, did you (1) stop practicing one or more sports to focus on a main sport?; (2) practice a single sport more than eight months per year?; and (3) consider that your main sport was more important than other sports? Respondents who said ‘yes’ to all three questions, were considered in the group of early sport specialization.

### 2.2. Procedure

Six secondary schools were contacted and five were willing and able to participate within the timeframe of the study (February 2020). Schools were selected via personal connections of the research team, and school directors were individually contacted. Two weeks before the data collection day, parents of all students of the selected classes in the second and third grade were informed about the study via an information letter, distributed by the class teacher. Parents had the opportunity to prevent their child from participating in the study (i.e., passive parental consent). One parent used this opt-out possibility for their child. On the day of data collection, two researchers were present on site. They verbally informed all students in the selected class groups about the study. The researchers explained the informed consent procedure and provided an information sheet containing a unique code which they entered at the start of the online survey, after giving their online consent to participate. Parents had the opportunity, until two weeks after data collection, to withdraw the responses of their child, by e-mailing the researchers with the unique code linked to their child’s participation. None of the parents used this option. The data collection took place during a regular class period of 50 min, which allowed enough time for a briefing, completion of the survey, and a debriefing. During data collection, the two researchers were present in the classroom and available for technical and emotional support. The school’s designated welfare officer was on stand-by in case any participant needed support. Approval for the research protocol was obtained from the Social and Societal Ethics Committee of the KULeuven (file number G-2019 11 1827).

A total of 801 students started the survey. Those who indicated they had never been involved in organized sport (*n* = 32; 4.0%) were not permitted to continue the questionnaire. All included respondents had participated in organized sport, with 77.6% (*n* = 597) indicating that they were currently participating in sport and 22.4% (*n* = 172) indicating that they had previously participated. Students in second and third grade of secondary schools in Belgium are usually between 14 and 18 years old. Some young people take a bit longer to finish high school, which explains the inclusion of fourteen respondents aged 19, three aged 20, and two aged 21 in this sample. Furthermore, one young person of 13 years old was included, probably because he or she skipped a year in school. Socio-demographic and sport specific characteristics of the respondents were also collected, and are reported in the Results section.

*Statistical procedure*. Frequency analysis and Chi Square tests were used to compare the proportion of IV exposure (low threshold) between boys and girls. The level of exposure to each type of IV was calculated based on the mean frequency score (0 = never; 1 = one or two times; 2 = three to ten times; 3 = more than 10 times), for all items in the specific IV-subscale, on the condition that at least 75% of the items in the scale were completed. As a result, scale scores ranged from 0 to 3. To examine factors potentially associated with elevated IV-exposure, a multiple linear regression model was used (see Figure S1 in Supplementary Materials). Independent variables in the model were: age, sex, ethnicity, sexual orientation, disability sports, weekly training hours (+16 h), sport level, early sport specialization, and type of sport (individual sports, team sports, both). Each type of IV was used as dependent variable: psychological violence—neglect, instrumental violence, physical violence, sexual violence, IV by a peer athlete, IV by a coach, and IV by a parent in the context of sport. The level of partial missing data was very low (maximum 2.3%). Following Schafer’s assertion [19] that a missing rate of 5% or less is inconsequential for statistical inferences, missing data were not treated. Data were analyzed using SPSS version 27.0. Significance levels were set at an Alpha level of 0.05.

## 3. Results

### 3.1. Description of the Sample

The sample of survey respondents consisted of 66% males (*n* = 507), 33% females (*n* = 256), and 0.7% (*n* = 5) young people who indicated to be intersex. The average age of the respondents was 15.9 years old (sd = 1.3) and ranged from 13 to 21 years.

In terms of the sports respondents engaged in, soccer (European football) was the most practiced primary sport in this sample (*n* = 233; 32.0%). Other popular primary sports in this sample were fitness (*n* = 48; 6.6%), dance (*n* = 46; 6.3%), martial arts (*n* = 43; 5.9%), and tennis (*n* = 41; 5.6%). For females, dance (*n* = 42; 16.4%), tennis (*n* = 22; 8.6%), and gymnastics (*n* = 18; 7.0%) were the most practiced sports. For males, soccer (*n* = 224; 44.2%) was the most practiced sport by an overwhelming margin, followed by martial arts (*n* = 36; 6.9%), and cycling (*n* = 31; 6.1%). Most respondents (*n* = 323; 42.0%) were active in individual sport(s) only, with 26.3% participating in team sports only (*n* = 202), and the remaining 19.5% (*n* = 150) participating both in team and individual sports.

The majority of respondents were members of a sports club (*n* = 581; 75.6%), but others (also) practiced sport through commercial sport providers (e.g., fitness centers) (*n* = 181; 23.5%), informal groups (*n* = 174; 22.6%), alone (*n* = 166; 21.6%) or in organized extracurricular sports or sport camps (*n* = 122; 15.9%). About 8.3% (*n* = 64) participated (exclusively or not) in sport for people with a disability. The average age at which these young people commenced organized sport was 6 years old (sd = 3.0).

The athletes’ competition levels in this sample varied from recreational (*n* = 120; 15.9%) to local (*n* = 54; 7.1%), regional (*n* = 279; 36.9%), national (*n* = 236; 21.2%) and even international competitions (*n* = 67; 8.9%). The relatively high proportion of athletes competing internationally was due to the inclusion of one school with an elite athletes’ program. Of note, this school also had significantly more male respondents competing at the national and international level, compared to females (45.2% vs. 29.2%; Χ^2^ (4; *n* = 751) = 53.7, *p* < 0.001).

We further categorized 223 respondents (29.3%) as athletes with an early specialization profile in one specific sport. About 24% of the respondents invested less than five hours a week in sports and about 50% invested between 6 and 15 h. The remaining 25% of respondents practiced sports for 16 or more hours a week. More than half of the respondents (*n* = 434; 56.4%) had stayed overnight at training centers or camps.

### 3.2. Magnitude of IV

Only 94 athletes, or 14% of respondents, did not report any experience of IV in sport. Moreover, the majority of respondents reported more than one type of IV, with two thirds of all respondents reporting two or more types of IV (see Table 1). All types of IV were prevalent in this sample, ranging from 27% (sexual violence) to 79% (psychological violence and neglect). Male respondents reported significantly more physical violence, while females reported significantly more sexual violence. No sex differences were found for psychological-neglect and instrumental violence.

IV perpetrated by peer athletes was reported to the same degree as IV perpetrated by a coach (70%), while IV perpetrated by a parent in the context of sport was somewhat less common, but still prevalent (48%). There were no differences between male and female respondents’ experiences of IV. according to type of perpetrator.

The most common experiences with IV from peer athletes were types of psychological violence, such as being excluded from the group, having rumors or hurtful comments said about you, and receiving insults, threats or being humiliated (See Table S1 in Supplementary Materials). Each of these behaviors were experienced by at least 30% of the respondents. Violence from a coach was somewhat less prevalent, but, nonetheless, more than 20% of respondents in our sample had experienced being excessively criticized, rejected, excluded, shouted at, insulted or humiliated by their coach. Almost one in three male respondents reported that the coach threw objects at them. Irrespective of sex, the most common types of IV from a parent in sport were being excessively criticized and insulted or humiliated

### 3.3. Factors Associated with Elevated IV Exposure

Table 2 shows the significant associated factors for each type of IV and perpetrator that were included in the regression model. The predictors collectively explained between 1 and 12% of experienced violence of variance 1% (sexual), over 8% (physical), 10% (psychological-neglect) and 12% for instrumental violence. In terms of perpetrators, the models explained 3% of the variance in athlete-perpetrated violence, 7% in coach-perpetrated violence and 10% in parent-perpetrated violence.

Athletes with a non-heterosexual orientation reported more violence experienced from other peers, as well as higher overall rates of psychological violence and neglect, and instrumental violence. Respondents who participated in disability sport (it was not specified whether or not the respondent actually had a disability) reported more psychological violence and neglect. Athletes who trained 16 h or more per week, reported higher IV-exposure levels for all types of violence from all types of perpetrators. This predictor was strongest for instrumental violence and violence from a parent in the context of their sport. A higher sport level was associated with more IV from a coach, while early specialization was associated with more IV from a parent. Both sport level and early specialization led to more psychological violence and neglect, and instrumental violence. Athletes who were only active in a team or an individual sport, reported lower levels of physical and peer-to-peer violence compared to athletes who participated in both types of sport.

## 4. Discussion

This study aimed to describe the magnitude of IV in sport in a sample of young students, aged 13 to 21 years old, in five secondary schools in Flanders, Belgium, using the VTAQ, the most up-to-date and validated tool to measure violence towards young athletes [17]. The results showed that all types of IV are present in Flemish youth sport. The combined group of psychological violence and neglect was the most prevalent form of IV, reported by four out of five respondents. Both physical and instrumental violence were reported by half of the respondents and sexual violence was reported by one in four respondents. The most common perpetrators were peers and coaches (both 70%), and, to a lesser extent, parents in the context of sport (48%). Due to these high numbers, it is not surprising that the majority of respondents reported two or more types of IV experienced in sport.

The reported magnitude of all forms of IV were almost double those of the first Belgian prevalence study [10]. While it is possible that the magnitude has increased during this timeframe, we should also consider other possible explanations for the discrepancy. First, there is a difference in the tools used. In the current study, we used a recently developed, sensitive, comprehensive tool that contains specific on field and off field violent behaviors in the context of sport. In contrast, the original Belgian study consisted of fewer individual items describing IV and did not include any pertaining to on field violence. Research has shown that, in survey instruments, the more items that are presented to operationalize a concept, e.g., sexual harassment, the higher the reported prevalence rates are [12]. Second, the current study sampled young people who are currently or were very recently active in organized sport, while the first Belgian study used a retrospective design with a sample of adults aged 18 to 50. While a loss of memory related to their sport experiences over time possibly influenced the magnitude of the 2016 study, that is much less likely to have been a factor in the present study. Thirdly, the questions in the VTAQ are formulated in a neutral way, avoiding loaded terms like ‘abuse’ or ‘unwanted’, allowing for capturing experiences that were not recognized by the participants as abusive or violent [15]. Lastly, due to increased attention to harassment and abuse in and outside sport, covered in the media and highly debated in sport and general society, we hypothesize that young people are now more aware about unwanted behaviors and could potentially more easily recognize, acknowledge, and report such experiences.

The current findings are also in line with other studies using the VTAQ. In our study, psychological violence and neglect was high (79%), but at similar levels to both the Canadian (81%) and the Australian (76%) study [9,20]. Physical violence in our study (54%) was midway between the Canadian (40%) and Australian sample (66%), and sexual violence measured in our study (27%) was close to Canada (28%), but well below what was reported in Australia (38%). When comparing these findings, we noted that the Australian study used a self-selecting online convenience sample of adults in a retrospective design, which resulted in a different selection bias, compared to the Canadian and current studies.

In terms of sex differences, our results are also in line with the original Canadian study: females reported more psychological violence and neglect, while males reported more physical violence. Scholars have explained that male-dominated sports are more vulnerable for hypermasculine norms and values, including high normalization of physically violent behaviors between and towards athletes [21]. Regarding instrumental violence, while there were no sex differences, this type of violence was linked to other aspects (i.e., training hours). This suggests that all athletes (regardless of sex) are potentially susceptible to experiencing instrumental violence in higher performance sport cultures that privilege a ‘winning at all cost’ mentality [22]. Regarding sexual violence, our study found higher exposure in female athletes, which is in line with most research on sexual violence in and outside sport, but was not found in the Canadian study using the VTAQ [9]. The Canadian sport context seems to be less conducive for sexual violence towards girls, compared to the Belgian context. This could be related to different prevention efforts towards women and girls in sport, but remains to be investigated in future research.

When exploring which factors are associated with elevated IV-exposure, the most significant predictors were the number of weekly training hours and participation in different sport types (both individual and team sports). Athletes who spent 16 h or more in their sport on a weekly basis reported significantly more IV in all types and from all perpetrators. Athletes who participated in both individual and team sports reported more physical and peer-to-peer violence, compared to athletes involved exclusively in individual or team sports. Spending more time in sport contexts increases the possibility of being exposed to IV. It is likely that children who spend more hours involved in sport, also develop closer relationships with others in these contexts, which might increase the risk of exposure to IV [23]. The impact of weekly training hours was high in violence perpetrated by a parent, suggesting that parents play an important, and sometimes ambiguous, role in the safety of their child’s sport participation. At the same time, the respondents’ weekly training hours was positively associated both with competition levels and with exposure to instrumental violence, showing that these cultures are more present in high performance sport [22]. The same, although less significantly, was found in the Canadian and Australian studies. Qualitative studies have shown that performance-related abuse is, to a great extent, normalized in sport by athletes, coaches, entourage and parents [7,24,25]. It is likely that this normalization of this type of violence is highest in environments where the expectations and investments are high, such as in elite sport, but this has yet to be explored in depth.

Compared to all other types of violence, experience of sexual violence is the least predicted by the factors in our model. This indicates that other personal, interpersonal and societal characteristics beyond those currently included in the model are more defining for the risk of sexual violence. Predicting or explaining individual risk for sexual violence can thus only be improved when other characteristics, e.g., prior victimization, a non-nuclear family structure, social isolation, health problems, (lack of) familial and social support can be included in the analysis [26]. It is also likely that predictors for non-contact sexual harassment are different from those for contact forms of sexual violence, making it worthwhile to investigate these forms separately in future studies.

Interpretation of these findings should be in light of several limitations. The sample was not representative for the total population of young athletes in Flanders. On the other hand, there was very little chance of a self-selection bias in this sample, since the school administration selected the class groups that could participate in the study and (almost) all students from those classes participated in the survey. However, this sampling also resulted in the inclusion of more males compared to females, as well as a high proportion of young (mostly male) athletes competing at the international level. Both male sex and higher competition level are associated with experiencing more IV, which should also be taken into consideration when interpreting these results. As this study was not representative for sport disciplines, it is not possible to analyze differences in IV exposure between sport disciplines. Future research with representative samples from multiple disciplines could investigate sport-specific risk factors and exposure levels. Since respondents were invited to complete the online survey in the classroom on computers or tablets, and were sometimes sitting in close proximity to their classmates, it is possible that some of them tended towards selecting socially desirable responses. This limitation was eliminated as much as possible by allowing as much space as possible and by randomizing the questionnaire parts between all respondents. The ethical consideration of proximity of the researchers, teacher and school counsellor during data collection outweighed the risk of socially desirable responses due to lack of distance between respondents. Lastly, because of the cross-sectional design, we could not determine the chronology, or synchronicity, between overlapping experiences of different types of IV.

## 5. Conclusions

The findings of this study show that all types of IV are present in youth sport. These results can serve as a baseline measurement to evaluate current and future initiatives to safeguard young athletes in Flanders. Future research should consider further investigating the co-occurrence and chronology of different types of IV and the combination of athlete, other sport stakeholders and context characteristics (e.g., sport organization culture and safeguarding policies) to map risk and protective factors for IV in sport more precisely. While IV in sport is neither justifiable nor inevitable [27], studies continue to find high rates of IV across varying countries and sport disciplines. Based on our findings, instrumental forms of violence, for the purpose of ‘performance enhancement’ deserve special attention in research, policy and practice. This study will help to raise awareness in sport leadership, public decision makers, the sports community, and the general public. The findings demonstrate that some forms of IV are instrumental and linked to the sport culture, showing the need for safeguarding policies addressing both individual as well as organizational and cultural violence enablers. Only if all stakeholders are convinced that a happy and healthy athlete is a well-performing athlete, can training regimes and sport cultures allowing for violence and abuse be eradicated.

## Figures and Tables

**Table 1:** Frequencies of each type of IV and the number of different types experienced.

	Total *n* (%)	Girls *n* (%)	Boys *n* (%)	Chi Square	*p*
**Type of IV** ^a^					
Psychological violence and neglect	579 (78.8)	199 (80.6)	380 (77.9)	0.714	0.398
Physical	396 (53.5)	102 (41.0)	294 (59.9)	23.759	<0.001
Instrumental	376 (50.2)	121 (47.6)	255 (51.5)	1.009	0.315
Sexual	201 (26.8)	81 (32.0)	120 (24.2)	5.221	0.022
Any type of IV	603 (86.5)	205 (86.1)	398 (86.7)	0.045	0.833
All types of IV	11 (1.6)	3 (1.3)	8 (1.7)	0.235	0.628
**Number of types** ^a^					
No	94 (13.5)	33 (13.9)	61 (13.3)	9.623	0.047
One type of IV	136 (19.5)	59 (24.8)	77 (16.8)		
Two types of IV	172 (24.7)	59 (24.8)	113 (24.6)		
Three types of IV	183 (26.3)	49 (20.6)	134 (29.2)		
Four types of IV	112 (16.1)	38 (16.0)	74 (16.1)		
**Perpetrator of IV** ^a^					
IV by an athlete	527 (70.1)	184 (72.7)	343 (68.7)	1.275	0.259
IV by a coach	512 (70.5)	171 (68.7)	341 (71.5)	0.623	0.430
IV by a parent	352 (47.8)	119 (48.2)	233 (47.6)	0.026	0.872

^a^ low threshold score (01)—if any of the items is 1, the category is 1.

**Table 2:** Regression for type of IV and IV per perpetrator.

				Predictors (stdβ)							
	Adjusted *R*^2^	*F*	*p*	Sex ^b^ (Male)	Sexual Orientation (Heterosexual)	Disability Sports	Weekly Training Hours (16+ Hours)	Sport Level	Early Specialization	Only Individual Sports	Only Team Sports
**Type of IV ^a^**											
Psychological-neglect	0.099	8.090	<0.001	−0.098 *	−0.078 *	0.074 *	0.177 **	0.084 *	0.130 **		
Physical	0.077	5.345	<0.001	0.138 **			0.144 **			−0.177 **	−0.193 **
Instrumental	0.123	10.098	<0.001		−0.083 *		0.222 **	0.111 **	0.092 *		
Sexual	0.013	1.845	0.044	−0.082 *			0.079 *				
**Perpetrator of IV ^a^**											
IV by an athlete	0.030	3.005	0.001		−0.095 *		0.118 **			−0.134 *	−0.156 *
IV by a coach	0.072	5.994	<0.001				165 **	0.093 *			
IV by a parent	0.098	8.052	<0.001				0.201 **		0.146 **		

* *p* < 0.05, ** *p* < 0.01. ^a^ total score based on means of all items in this type of IV. ^b^ respondents who indicated be intersex, were excluded from this analysis. Predictors in the model: Age, sex, ethnicity, sexual orientation, disability sports, weekly training hours (+16 h), sport level, early specialization, type of sport. Age and ethnicity were no significant predictor in any of the models, and were therefor not included in this table.

## Data Availability

The authors will make the dataset generated during this study available.

## Supplementary Materials

The following supporting information can be downloaded at: https://www.mdpi.com/article/10.3390/ijerph191811745/s1, Table S1: Frequency of the experience with IV from peer athletes, coaches and parents in the context of sport (VTAQ), ordered by frequency in the total sample. Figure S1: Visual representation of the regression model for factors related to IV exposure.
