# Supplementary material for: Interpersonal Violence in Belgian Sport Today: Young Athletes Report
Source: Int J Environ Res Public Health. 2022 Sep 17;19(18):11745. doi: 10.3390/ijerph191811745 (PMC9517528; doi:10.3390/ijerph191811745)
Supplement: Supplementary file 1 [file ijerph-19-11745-s001.zip › ijerph-1889595-supplementary.pdf]

Supplementary Materials

Figure S1. Visual representation of the regression model for factors related to IV exposure

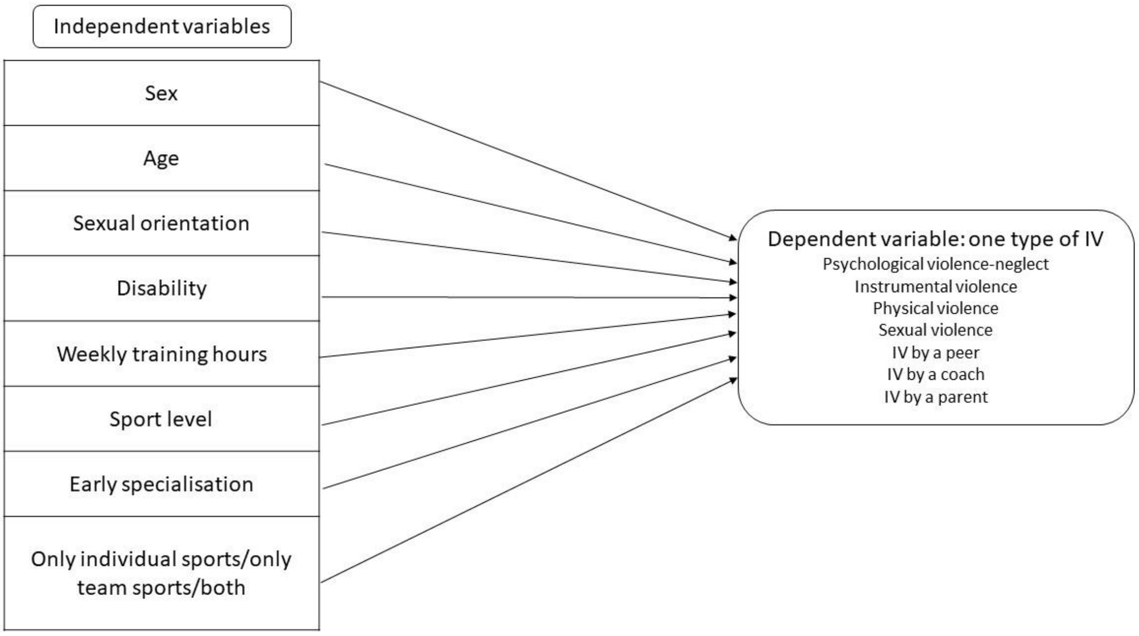

Table S1. Frequency of the experience with IV from peer athletes, coaches and parents in the context of sport (VTAQ), ordered by frequency in the total sample

Item	Summary of item	Total sample		Female respondents		Male respondents	
		n	%	n	%	n	%
A2	Gossip, humiliations	294	38.4	113	44.1	180	35.5
A4	Threats and insults	285	37.2	88	34.4	197	38.9
A1	Exclusion	279	36.4	119	46.5	158	31.2
C12	Excessive critiques	260	33.9	81	31.6	178	35.1
C2	Thrown object at you	192	25.1	44	17.2	147	29.0
C11	Insults, humiliations	189	24.7	68	26.6	119	23.5
P11	Excessive critique	189	24.7	59	23.0	130	25.6
A5	Hit, pushed during competition	151	19.7	29	11.3	121	23.9
C13	Thrown out or excluded	148	19.3	66	25.8	82	16.2
C22	Forced to participate with injury (against medical advice)	144	18.8	47	18.4	96	18.9
C7	Objects thrown around	138	18.0	39	15.2	98	19.3
C17	Forced additional trainings	138	18.0	41	16.0	97	19.1
C18	Forced to train while injured	127	16.6	50	19.5	76	15.0
A3	Damage belongings	125	16.3	30	11.7	93	18.3
C16	Ignored or ignorant	120	15.7	59	23.0	60	11.8
C19	Forced to complete too difficult movements	120	15.7	52	20.3	67	13.2
A7	Humiliating sexual comments	119	15.5	47	18.4	71	14.0
A6	Hit, pushed outside competition	118	15.4	26	10.2	90	17.8
P10	Insult, humiliate	118	15.4	42	16.4	76	15.0
C15	Asked to limit social contacts	117	15.3	46	18.0	70	13.8
C9	Not intervened when you injured an opponent	108	14.1	16	6.3	90	17.8
C8	Obliged to injure an opponent	90	11.7	10	3.9	78	15.4
P16	Asked to limit social contacts	90	11.7	38	14.8	52	10.3
P3	Hit with hand	89	11.6	24	9.4	65	12.8
C1	Being pushed	88	11.5	17	6.6	70	13.8
A8	Unacceptable sexual behavior	83	10.8	43	16.8	40	7.9
P2	Thrown an object at you	76	9.9	13	5.1	63	12.4
P14	Ignore or being ignorant	75	9.8	27	10.5	48	9.5
C26	Sexual comments	70	9.1	36	14.1	33	6.5
C3	Hit with hand	68	8.9	13	5.1	54	10.7
P21	Forced participation with injury (against doctor's advice)	68	8.9	23	9.0	45	8.9
C25	Asked to stop school	66	8.6	30	11.7	36	7.1
P1	Being pushed	65	8.5	15	5.9	50	9.9
C20	Forced to lose weight	60	7.8	21	8.2	39	7.7
P7	Objects thrown around	57	7.4	16	6.3	41	8.1
P17	Forced to train while injured	56	7.3	22	8.6	34	6.7
P15	Forced additional trainings	50	6.5	15	5.9	35	6.9
P12	Thrown out or excluded	46	6.0	21	8.2	25	4.9
C4	Hit with fist	45	5.9	8	3.1	36	7.1
C27	Sexual non-contact behavior	43	5.6	29	11.3	14	2.8
P8	Obliged to injure an opponent	40	5.2	7	2.7	31	6.1

C23	Not intervene in doping use	34	4.4	9	3.5	24	4.7
P18	Forced to complete too difficult movements	31	4.0	14	5.5	17	3.4
C10	Threat to abandon	30	3.9	8	3.1	21	4.1
P4	Hit with fist	29	3.8	2	0.8	27	5.3
P19	Forced to lose weight	29	3.8	11	4.3	18	3.6
C29	Sexual conversations	26	3.4	9	3.5	15	3.0
P9	Thread to abandon	26	3.4	10	3.9	16	3.2
P25	Deny medical care	24	3.1	11	4.3	13	2.6
C6	Grabbed by the throat	22	2.9	2	0.8	19	3.7
C33	Touched other body parts	18	2.3	11	4.3	7	1.4
P22	Not interfere with weight loss	18	2.3	5	2.0	13	2.6
C5	Hit with hard object	17	2.2	6	2.3	10	2.0
P6	Grabbed by the throat	16	2.1	4	1.6	12	2.4
C28	Watched you while undress or masturbate	15	2.0	6	2.3	8	1.6
C34	Sexual kissing	15	2.0	4	1.6	11	2.2
P5	Hit with hard object	15	2.0	1	0.4	14	2.8
P24	Asked to stop or pause school	15	2.0	5	2.0	10	2.0
A9	Forced sexual contact	14	1.8	9	3.5	5	1.0
P13	Locked up	14	1.8	1	0.4	13	2.6
C31	Exhibitionism	12	1.6	2	0.8	9	1.8
C14	Locked up	11	1.4	2	0.8	9	1.8
C21	Forced to use doping	11	1.4	1	0.4	9	1.8
C24	Not intervene in doping use	10	1.3	1	0.4	8	1.6
C36	Sexual penetration	10	1.3	0	0.0	9	1.8
C35	Attempted sexual penetration	9	1.2	1	0.4	7	1.4
P23	Not intervene in doping use	9	1.2	0	0.0	9	1.8
C32	Touched genitals	8	1.0	0	0.0	7	1.4
C30	Filmed during sexual behavior	6	0.8	1	0.4	4	0.8
P20	Forced to use performance-enhancing drugs	6	0.8	0	0.0	6	1.2

Notes.
Items starting with an A relate to violence from a peer athlete.
Items starting with a C relate to violence from a coach
Items starting with a P relate to violence from a parent
The total number of respondents consist of male and female respondents, as well as those we did not identify as male or female and those not disclosing their biological sex.
